# Ensemble learning with speaker embeddings in multiple speech task stimuli for depression detection

**DOI:** 10.3389/fnins.2023.1141621

**Published:** 2023-03-23

**Authors:** Zhenyu Liu, Huimin Yu, Gang Li, Qiongqiong Chen, Zhijie Ding, Lei Feng, Zhijun Yao, Bin Hu

**Affiliations:** ^1^Gansu Provincial Key Laboratory of Wearable Computing, School of Information Science and Engineering, Lanzhou University, Lanzhou, China; ^2^Tianshui Third People’s Hospital, Tianshui, China; ^3^Second Provincial People’s Hospital of Gansu, Lanzhou, China; ^4^Affiliated Hospital of Northwest Minzu University, Lanzhou, China; ^5^Department of Psychiatry, Beijing Anding Hospital of Capital Medical University, Beijing, China

**Keywords:** depression detection, Resnet x-vectors, speaker embeddings, ensemble learning, speech task stimuli

## Abstract

**Introduction:**

As a biomarker of depression, speech signal has attracted the interest of many researchers due to its characteristics of easy collection and non-invasive. However, subjects’ speech variation under different scenes and emotional stimuli, the insufficient amount of depression speech data for deep learning, and the variable length of speech frame-level features have an impact on the recognition performance.

**Methods:**

The above problems, this study proposes a multi-task ensemble learning method based on speaker embeddings for depression classification. First, we extract the Mel Frequency Cepstral Coefficients (MFCC), the Perceptual Linear Predictive Coefficients (PLP), and the Filter Bank (FBANK) from the out-domain dataset (CN-Celeb) and train the Resnet x-vector extractor, Time delay neural network (TDNN) x-vector extractor, and i-vector extractor. Then, we extract the corresponding speaker embeddings of fixed length from the depression speech database of the Gansu Provincial Key Laboratory of Wearable Computing. Support Vector Machine (SVM) and Random Forest (RF) are used to obtain the classification results of speaker embeddings in nine speech tasks. To make full use of the information of speech tasks with different scenes and emotions, we aggregate the classification results of nine tasks into new features and then obtain the final classification results by using Multilayer Perceptron (MLP). In order to take advantage of the complementary effects of different features, Resnet x-vectors based on different acoustic features are fused in the ensemble learning method.

**Results:**

Experimental results demonstrate that (1) MFCC-based Resnet x-vectors perform best among the nine speaker embeddings for depression detection; (2) interview speech is better than picture descriptions speech, and neutral stimulus is the best among the three emotional valences in the depression recognition task; (3) our multi-task ensemble learning method with MFCC-based Resnet x-vectors can effectively identify depressed patients; (4) in all cases, the combination of MFCC-based Resnet x-vectors and PLP-based Resnet x-vectors in our ensemble learning method achieves the best results, outperforming other literature studies using the depression speech database.

**Discussion:**

Our multi-task ensemble learning method with MFCC-based Resnet x-vectors can fuse the depression related information of different stimuli effectively, which provides a new approach for depression detection. The limitation of this method is that speaker embeddings extractors were pre-trained on the out-domain dataset. We will consider using the augmented in-domain dataset for pre-training to improve the depression recognition performance further.

## 1. Introduction

Depression is a common and recurrent mood disorder accompanied by functional disability, significantly impacting the individual’s physical and mental health and daily activities (Spijker et al., 2004). More than 300 million people worldwide suffer from depression, equivalent to 4.4% of the world’s population (World Health Organization, 2017). The latest scientific brief shows a dramatic 25% increase in the global prevalence of anxiety and depression in the first year of the Coronavirus 2019 (COVID-19) pandemic (World Health Organization, 2022). At present, the diagnostic methods for depression detection mainly rely on psychiatrists and scales. The accuracy of diagnostic results is affected by subjective factors such as doctors’ clinical experience and whether patients can fully describe their physiological and psychological conditions.

On the other hand, in China, only 7.1% of depression patients who seek treatment in mental health institutions receive adequate treatment (Lu J. et al., 2021). The lack of medical resources leads to many patients being unable to see a doctor in time. Therefore, exploring objective and effective new techniques to identify depression has attracted much attention. Researchers have focused on seeking objective biological markers [i.e., gut hormones (Rajkumar, 2021)], physiological markers [i.e., EEG (Cai et al., 2020)] and eye movement (Shen et al., 2021), and behavioral markers [i.e., speech (Othmani et al., 2021) and facial expressions (Guo et al., 2021)] to aid in the diagnosis of depression. Among these markers, speech signal has become an important research direction for auxiliary diagnosis of depression due to its advantages of acquisition, non-invasion, non-disturbance, low cost, and a large amount of information.

Depression patients are typically sluggish (Beck and Alford, 2009), with longer pauses (Szabadi et al., 1976; Greden and Carroll, 1980) and a lack of rhythm (Alpert et al., 2001). The research showed that the percentage of pause time, the standard deviation of fundamental frequency distribution, the standard deviation of fundamental frequency change rate, and speech speed are correlated with the clinical status of patients with depression (Nilsonne, 1987). There is a strong correlation between speed, percent pause, pitch variation, and scale score (Cannizzaro et al., 2004). Depressed people treated and improved had more significant variation in pitch cycles, fewer pauses, and faster speech (Mundt et al., 2007). Thus, depressed people and healthy people have different pronunciations.

In order to make full use of the influence of speech tasks with different scenes and different emotional stimuli on speech of depressed patients and normal subjects, we designed a multi-task ensemble learning method with speaker embeddings in our depression speech dataset containing 9 speech tasks, and proved the effectiveness of this method from the accuracy, F1-D and F1-H.

The organization of the paper is as follows. The second section briefly reviews some related studies. The two datasets used in this paper are introduced in the third section. Next, the fourth section describes the multi-task ensemble learning method using speaker embeddings for depression recognition proposed in this study. Afterward, in the fifth section, the experimental results are presented. Finally, the conclusions and future works are summarized in the sixth section.

## 2. Related works

At present, there have been many approaches for depression recognition based on speech processing. Searching for effective acoustic features has always been an important research direction. Manual features such as spectral, source, prosodic, and formant features are commonly employed when analyzing depression and suicidality (Cummins et al., 2015). Moreover, these features are also regarded as inputs to deep neural networks (Lang and Cui, 2018; Lu X. et al., 2021). Studies have shown that the advanced features generated by MFCC feeding into the Short Long-Term Memory (LSTM) can preserve information related to depression (Rejaibi et al., 2022). PLP, and MFCC, called the low-level descriptors, are used to train the multiple classifier systems (Long et al., 2017). The input of the network model is a 3D feature made up of FBANK, the first-order and second-order differences to use the information in speech signals entirely (Wang et al., 2021). The findings of the aforementioned study illustrate that MFCC, PLP, and FBANK as front-end features can refine enough speech details.

Speaker embeddings such as i-vectors, d-vectors, and x-vectors have shown their superiority in speaker recognition (Variani et al., 2014; Wang et al., 2017), and depression detection (Egas-López et al., 2022). Scholars have found that speaker embeddings cannot only solve the variable length problem of frame-level features but also encode the speaker identity and the speech content to a large extent (Wang et al., 2017). In addition, speaker embeddings we extracted are based on the pre-trained speaker recognition model, which can be used for depression recognition tasks. The i-vectors, the low-dimension compact representations, were first proposed for speaker verification (Dehak et al., 2010). Afterward, the i-vector framework was widely applied in speaker recognition (Kanagasundaram et al., 2012), emotion recognition (Vekkot et al., 2019), Alzheimer’s disease (AD) detection (Egas López et al., 2019), Parkinson’s disease (PD) detection (Garcia et al., 2017), and depression detection (Cummins et al., 2014; Rani, 2017; Afshan et al., 2018; Mobram and Vali, 2022). Furthermore, the correlation between MFCC i-vectors and MFCC features has been determined, and the effectiveness of i-vectors has been examined in diagnosing major depressive disorder (MDD) (Di et al., 2021). A comparison of various i-vectors based on spectral features, prosodic features, formants, and voice quality for clinical depression detection during the interview discovered that spectral feature i-vectors gained the highest accuracy in distinguishing between the speech of depressed and control (Xing et al., 2022). I-vectors can limit speaker and channel variability, which helps the model focus more on depression detection. With the development of the embedding technique, Deep Neural Network (DNN) embeddings, fixed-dimensional speaker embeddings extracted from a feed-forward DNN outperformed i-vectors for text-independent speaker verification on short speech segments (Snyder et al., 2017). X-vectors, the new state-of-the-art speaker embeddings, have been applied in speaker recognition (Snyder et al., 2017, 2018, 2019; Garcia-Romero et al., 2019). The encoder networks of x-vectors include the following categories: TDNN (Waibel et al., 1989), Extended TDNN architecture (E-TDNN) (Snyder et al., 2019), the factorized TDNN (F-TDNN) with skip connections (Povey et al., 2018), and Resnet 2D (He et al., 2016). Experiments show that x-vectors can capture spoken content and channel-related information (Raj et al., 2019). Furthermore, the TDNN x-vectors or F-TDNN x-vectors based on MFCC have demonstrated better performance than PLP i-vectors for the automatic detection of PD (Moro-Velazquez et al., 2020). Besides, the x-vector technique has been used as an advanced method for emotion recognition (Pappagari et al., 2020b), AD detection (Pappagari et al., 2020a), and depression detection (Dumpala et al., 2021, 2022; Egas-López et al., 2022). Consequently, depression detection is carried out in this study using the x-vector approach with the i-vector framework as the baseline.

One unavoidable problem is that the amount of depression data limits that model training. Publicly available and commonly used depression speech datasets are the Audio-Visual Emotion Recognition Challenge and Workshop (AVEC) 2013 (Valstar et al., 2013), including 340 video clips from 292 subjects, and AVEC 2014 (Valstar et al., 2014), including 150 files of 84 speakers. DNN trained on such data would lead to under-fitting; consequently, the classification result needs to be more convincing. One workable solution to the above problem is to pre-train a model on extensive data followed by leveraging the model’s knowledge to downstream tasks [e.g., speaker recognition (Snyder et al., 2018), PD detection (Moro-Velazquez et al., 2020), depression detection (Zhang et al., 2021)]. Primarily, results in Zhang et al. (2021) showed that the larger out-domain (e.g., speech recognition) dataset for audio embedding pre-training generally improves performance better than the relatively little in-domain (depression detection) dataset. Therefore, we pre-trained speaker embedding extractors on CN-Celeb (Fan et al., 2020), a large-scale Chinese speaker recognition dataset, followed by extracting corresponding embeddings on our Chinese depression speech dataset.

The method of training models with classification algorithms has occurred frequently in depression detection. SVM and RF were used for depression classification not only on low-level descriptors (LLD) and related functionals in Tasnim and Stroulia (2019) but also on i-vectors in Xing et al. (2022). On the other hand, the results of Saidi et al. (2020), comparing the baseline CNN model with the model combining CNN and SVM, have shown that the SVM classifier improved the classification accuracy. An exploratory study (Espinola et al., 2021), which compared experimental results of MLP, Logistic Regression (LR), RF, Bayes Network, Naïve Bayes, and SVM with different kernels, concluded that RF provided the highest accuracy among all classifiers for MDD detection. Therefore, SVM and RF were preferred as classification algorithms to evaluate speaker embeddings’ performance in our study comprehensively.

There have been studies showing that there are differences between depressed and normal subjects’ speech under different speech task stimuli. The collection of spontaneous and read speech from 30 depressed and 30 control subjects was used to extract acoustic features (Alghowinem et al., 2013). DEPression and Anxiety Crowdsourced corpus (DEPAC) (Tasnim et al., 2022), which has a diversity of speech tasks (Phoneme fluency, Phonemic fluency, Picture description, Semantic fluency, and Prompted narrative), has been published recently as a depression and anxiety detection corpus. Furthermore, the classification results in Long et al. (2017) based on the corpus of three speech types (reading, picture description, and interview), each of which corresponds to three emotional valences (negative, neutral, and positive), showed that speaking style and mood had a significant influence on depression recognition. From the theory of ensemble learning, combining multiple learners makes a whole’s generalization ability usually much more robust than a single learner (Zhou, 2021). Also, multiple speech modes with different affective valence are natural learners. As a result, this study combined the information of nine speech tasks under multiple scenes and emotional valences using the ensemble learning method to improve the depression recognition ability of the model.

The proposed depression detection system was based on the speaker embedding framework and a multi-task ensemble learning approach. The whole process was divided into two stages. The first stage is the process of pre-training speaker embedding extractors. Nine speaker embedding extractors that differed in the front-end features and framework were trained on CN_Celeb. Three front-end feature sets contained MFCC, PLP, and FBANK. Three embedding frameworks contained i-vector, TDNN, and Resnet. In this stage, each speaker embedding extractor could change frame-level features of different lengths into fixed lengths and, more importantly, overcome the challenge of insufficient depression data volume. The second stage is to extract speaker embeddings of the depression dataset and make further classification. The same front-end features were extracted for the depression data of nine tasks, and we obtained the corresponding speaker embeddings using the pre-trained extractors. The depression classification percentage of nine utterances from one subject attained by the SVM classifier were aggregated into integrated features. The final results were then obtained using MLP based on the new features.

The main contributions of this paper are as follows:

1. The speaker embedding extractors were pre-trained on the large-scale out-domain dataset to alleviate the problem of insufficient depression data for depression recognition.
2. We have proved that based on MFCC, PLP, and FBANK, Resnet x-vectors, which are first used to detect depression, outperform TDNN x-vectors, and i-vectors.
3. In the depression detection task, interview speech caught more acoustic differences between depressed and normal subjects than picture description speech. Neutral stimuli performed better compared to positive and negative stimuli.
4. The effectiveness of our multi-task ensemble learning approach was verified on multiple speaker embeddings. Moreover, our multi-task ensemble learning method with Resnet x-vectors can effectively identify depressed patients.

## 3. Database

Two speech corpora were employed in this study: the first, CN-Celeb, is an extensive Chinese speaker recognition dataset collected ‘in the wild’ for training i-vector, TDNN x-vector, and Resnet x-vector extractors; the other, the depression speech dataset, is a corpus containing recordings from normal and depressed subjects and was utilized to extract speaker embeddings (i-vectors, TDNN x-vectors, and Resnet x-vectors) and to train back-end classifiers and multi-task ensemble learning models to evaluate their performance in automatic depression detection.

### 3.1. CN-Celeb

CN-Celeb (Fan et al., 2020) contains more than 130,000 utterances from 1,000 Chinese celebrities, covering 11 different speech scenarios. We chose CN-Celeb for three reasons: its large quantity, which is an indispensable part of the pre-trained model; the language of all recordings is Chinese, which is the same as that of the depression dataset; and its rich speech genres, some of which match the tasks of the depression dataset. Because the task type of the depression speech dataset used in this experiment is interview and picture description, which are all spontaneous speech, the average length of each utterance is longer than 10 s. Based on the comprehensive consideration of speech modes and average duration of each utterance, we select all the speech in the interview and speech scenes of CN-Celeb. The subset includes 67,718 utterances from 902 Chinese celebrities with a total length of 171.99 h. The interview scenario contains 780 subjects with 59,317 utterances and lasts 135.77 h. As for the speech genre, 8,401 utterances from 122 speakers were collected, with a length of 36.22 h. All of them were sampled at 16 kHz.

### 3.2. Depression speech database

We collected speech data from Beijing Anding Hospital, Lanzhou University Second People’s Hospital, and Tianshui Third People’s Hospital. All subjects were aged between 18 and 55, native Chinese speakers, and had a primary school education or above. The patients were required to meet DSM-IV criteria (American Psychiatric Association, 1994) with the Patient Health Questionnaire-9 (PHQ-9) (Kroenke et al., 2001) score of 5 or greater and not to have taken any psychotropic drugs during the first 2 weeks of enrollment. In comparison, the control subjects had no definite mental disorder diagnosis and regular mental activity. In order to obtain high-quality speech data, the experiment was conducted in a room with good sound insulation and no electromagnetic interference, and the ambient noise was ensured to be lower than 60 dB. For the purpose of avoiding the distortion of the voice data, a high-precision sound card and microphone were used. The recordings were saved in Waveform Audio File Format (WAV) with a sampling rate of 44.1 kHz and a sampling width of 24 bit. The preprocessing steps of speech signal mainly included pre-emphasis, frame segmentation, and endpoint detection.

This dataset followed two different experimental paradigms whose intersection contained 9 identical speech tasks, including six interview tasks and three picture description tasks with three emotions (positive, neutral, and negative). The specific tasks are listed in Table 1. With regard to the evaluation of the valence of interview questions, we recruited 33 volunteers to score the valence and arousal of these questions, respectively, and then divided them into three types according to the degree of pleasure: positive, neutral and negative. The face images displayed in the picture description scene were taken from the Chinese facial affective picture system (CAPS) (Gong et al., 2011), which contains 870 facial images of seven emotions: anger, disgust, fear, sadness, surprise, happiness, and calm. The evaluation is conducted from the three dimensions of pleasure, arousal, and dominance. We selected three female face images of happiness, calm, and sadness as the picture description materials of positive, neutral, and negative stimuli. After Voice Activity Detection (VAD) to all recordings, data from 536 subjects, including 226 normal subjects and 310 depressed subjects, were preprocessed and retained. Each participant contained nine speech segments. Details of the depression speech dataset used in this study are shown in Table 2, including the subject number, utterance number, age, PHQ-9 score, and the average duration of each utterance in the two groups.

**TABLE 1:** Details of nine tasks.

Task	Genres	Valences	Problems
Task1	Interview	Positive	If you have a vacation to travel, please describe your travel plans.
Task2	Interview	Positive	Please share what you think is a good memory and briefly describe the scene.
Task3	Interview	Neutral	How are you feeling these days? How does this affect your life?
Task4	Interview	Neutral	How is your health these days? How has it affected your life?
Task5	Interview	Neutral	How do you rate yourself?
Task6	Interview	Negative	Describe an event that caused you great pain.
Task7	Picture description	Positive	Describe the positive facial expression, and guess the reason for the expression.
Task8	Picture description	Neutral	Describe the neutral facial expression, and guess the reason for the expression.
Task9	Picture description	Negative	Describe the negative facial expression, and guess the reason for the expression.

**TABLE 2:** Details of subjects’ information.

Subject type	Gender	Subject numbers	Utterance numbers	Age mean (standard deviation)	PHQ-9 mean (standard deviation)	Utterance duration mean(s)
Depression	Male	142	1,278	37.03 (10.88)	14.49 (7.15)	20.74
	Female	168	1,512	38.23 (12.14)	14.85 (8.24)	
Normal	Male	119	1,071	36.00 (10.82)	1.47 (2.31)	15.50
	Female	107	963	33.36 (10.53)	1.42 (0.69)	

## 4. Methodology

**FIGURE 1:**
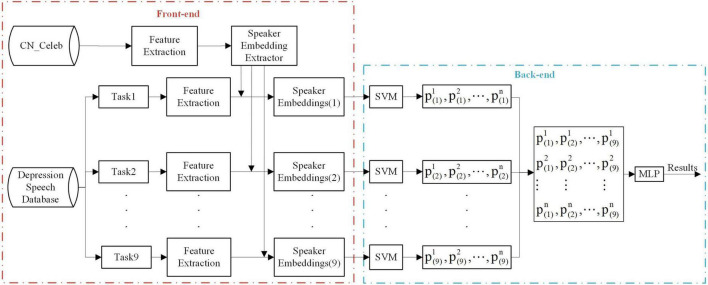
General methodology diagram of the proposed depression detection system. The acoustic feature could be MFCC, PLP, or FBANK. The speaker embedding extractor type can be i-vector, TDNN x-vector, and Resnet x-vector, and the speaker embedding type is derived from the extractor type. *n* represents the number of training subjects. The subjects are divided into 10 folds according to the 10-fold cross-validation rule, in which nine folds are used for training and onefold for testing.

The method proposed in this paper aims to improve depression classification performance using integrated learning combined with a pre-trained speaker embedding system and multiple speech task stimuli. Figure 1 shows a general block diagram of the depression detection system used in this study.

Firstly, the speech features are extracted from the preprocessed utterances (Section “4.1. Acoustic feature extraction”). Next, the speaker embedding extractors are pre-trained based on acoustic features of the out-domain dataset, and speaker embeddings of the multi-task in-domain dataset are extracted (Section “4.2. Speaker embedding extraction”). In order to take advantage of the effects of nine tasks, the multi-task integrated learning approach is carried out in Section “4.3. Multi-task ensemble learning method.” These are described in detail below.

### 4.1. Acoustic feature extraction

Three acoustic feature sets, including MFCC, PLP, and FBANK, were extracted from each utterance of both CN-Celeb and our depression speech dataset in this study. This process was implemented by Kaldi Toolbox (Povey et al., 2011). We used three kinds of frame-level representations: 60-dimensional MFCCs, 60-dimensional PLPs, and 60-dimensional FBANKs, all with a Hamming window, a frame-length of 25 ms, and a frame-shift of 10 ms.

Mel Frequency Cepstral Coefficients was proposed based on the acoustic characteristics of the human ear, which could be understood as the energy distribution of speech signals in different frequency ranges. MFCC often serves as a standard to fit i-vector models (Di et al., 2021) or x-vector models (Egas-López et al., 2022), or other deep network models (Rejaibi et al., 2022). The literature results convince us that MFCC can contribute to the training of speaker embedding systems.

PLP was proposed using the results obtained from human auditory experiments, and it was beneficial to extract anti-noise speech features. The results of Moro-Velazquez et al. (2020) comparing the i-vector extractors based on PLP and the x-vector extractors based on MFCC showed that the two systems had their advantages in PD detection. Therefore, we extracted PLP for a comparative study of depression recognition.

The response of the human ear to the sound spectrum is nonlinear. FBANK is a front-end processing algorithm that can improve speech recognition performance by processing audio similarly to the human ear. The literature demonstrated that FBANK was more effective than MFCC in x-vector training for the Escalation SubChallenge (José Vicente et al., 2021) and depression assessment (Egas-López et al., 2022). Consequently, FBANK was also extracted in this study for subsequent training of speaker embedding extractors.

### 4.2. Speaker embedding extraction

In this study, three frameworks were performed to train different types of speaker embedding extractors based on the acoustic characteristics of CN-Celeb. The task of pre-training is to improve the performance of speaker recognition. We transferred the knowledge learned in the pre-training process to the depression recognition task, that is, to retain the extractors obtained in the upstream task. We applied them to the speaker embedding extraction on phonetic features of the depression speech database. Note that i-vectors served as a classic baseline method without deep learning and TDNN x-vectors served as a DNN baseline. We focused on a new state-of-the-art speaker recognition method: the Resnet x-vectors in depression detection. The procedure of i-vector extraction was carried out using Kaldi. At the same time, the extraction of TDNN x-vectors and Resnet x-vectors was implemented on ASV-Subtools (Tong et al., 2021).

#### 4.2.1. I-vector extraction

The i-vector framework can map speech recordings of arbitrary duration to low dimensional space, and a compact representation of fixed length is obtained. Acquiring the Universal Background Model (UBM) is to train a diagonal covariance matrix and a full matrix on all training subjects’ speech data. UBM is a speaker–and channel-independent Gaussian Mixture Model (GMM), which can be regarded as the unified reference coordinate space of the training set. When initializing UBM, the number of Gaussian components, denoted as *C*, must be set. The *ith* (*i* = 1, 2, …, *C*)Gaussian component includes a weight (*w_i_*), a mean vector (μ_*i*_), and a covariance matrix (Σ_*i*_). Thus, the Gaussian mean supervector (*m*) of UBM can be obtained. Furthermore, the Gaussian mean supervector (*M*) of the utterance (*h*) from the speaker (s) is defined as follows:


(1) $$M_{s,h}=m+T\,\omega_{s,h}$$

Different from the two spaces (a speaker subspace and a session subspace) included in the Joint Factor Analysis (JFA) model, the total variability space (*T*), which contains the speaker and channel effects simultaneously, is employed in the i-vector model (Dehak et al., 2010). ω is the total variability space factor, and its maximum-a-posteriori (MAP) point estimate is the i-vector. After UBM training, the Baum-Welch statistics of each speaker in the training set are calculated, and *T* is iteratively estimated by the Expectation-Maximization (EM) algorithm. *M*_*s,h*_ is obtained using MAP adaptation followed by the estimation of i-vectors based on ω_*s*,*h*_. More details on the calculation of Baum-Welch statistics and i-vector estimation can be sought out in Dehak et al. (2010).

In this study, we set the number of Gaussian components as 256 and the i-vector dimension as 256.

#### 4.2.2. TDNN x-vector extraction

The TDNN x-vector approach provides a fixed-dimensional utterance-level representation by using a time-delay neural network and the features of variable-length speech. Extracting TDNN x-vectors contains several steps. Firstly, the TDNN architecture runs at the frame level. The current time step is represented by *t*. The input to the next frame-level layer is concatenated from the current frame and its context of past and future frames. Therefore, the next layer of frame-level representation condenses the temporal context information. As the network deepens gradually, the scope of the temporal context becomes wider. After three time-delay operations, one frame in the fourth layer corresponds to 15 frames in the context of the first layer. The stats pooling layer aggregates all the frames of the speech segment and calculates the mean and standard deviation. Finally, TDNN x-vectors are obtained in the segment-level layer.

Time delay neural network x-vectors and Resnet x-vector extractors were trained on the Pytorch framework. The speech utterances were divided into chunks of 200 frames, and we set the batch size as 64. Moreover, the dimension of TDNN x-vectors and Resnet vectors was 256, the same as that of i-vectors. The process of the Resnet x-vector extraction is detailed in Section “4.2.3. Resnet x-vector extraction.” We used a ralamb optimizer containing LookAhead and RAdam optimizer with Layer-wise Adaptive Rate Scaling (LARS). The learning rate was set to 0.001, attenuating every 400 steps and an attenuation factor of 0.7. The number of training sessions was 18.

#### 4.2.3. Resnet x-vector extraction

**FIGURE 2:**
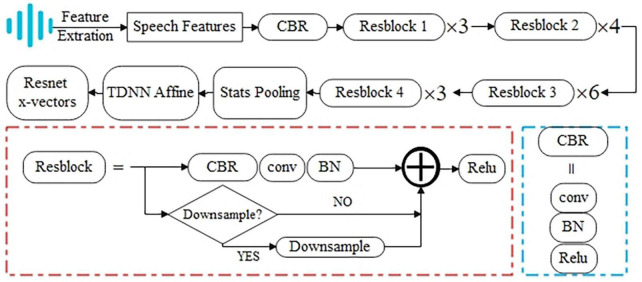
The block diagram of the Resnet x-vector extraction process.

Residual learning was proposed to simplify training for deeper networks (He et al., 2016). We followed the Resnet34 encoder described by Villalba et al. (2020) to train Resnet x-vector extractors. Figure 2 shows the block diagram of the Resnet x-vector extraction process. Specific architecture of the Resnet encoder is listed as Table 3. The repetition times of the four residual blocks are 3, 4, 6, and 3, respectively, and the number of residual block channels is gradually doubled from 32 to obtain deeper information. The dimension of acoustic features and the number of speech frames are denoted as *F* and *T*, respectively. When the stride is set to 2, the dimensions of *F* and *T* to the output are halved. Due to the addition operation in residual blocks, the input needs to be downsampled to ensure the same dimensions before adding. Finally, each speech segment can obtain Resnet x-vectors of fixed length after the average pooling layer.

**TABLE 3:** Resnet encoder architecture.

Layer	Input	Output	Down sample	Kernel	Stride	Channels	Blocks
Conv1	*F* × *T*	*F* × *T*	False	7 × 7	1	32	–
Resblock1	*F* × *T*	*F* × *T*	False	3 × 3	1	32	3
Resblock2	*F* × *T*	$\frac{F}{2}\times \frac{T}{2}$	True	3 × 3	2	64	4
Resblock3	$\frac{F}{2}\times \frac{T}{2}$	$\frac{F}{4}\times \frac{T}{4}$	True	3 × 3	2	128	6
Resblock4	$\frac{F}{4}\times \frac{T}{4}$	$\frac{F}{8}\times \frac{T}{8}$	True	3 × 3	2	256	3
Stats pooling	$\frac{F}{8}\times \frac{T}{8}$	$\frac{F}{4}\times 1$	–	–	–	256	–
TDNN affine	$\frac{F}{4}\times 1$	1 × 1	–	$\frac{F}{4}\times 1$	1	256	–

*F* is the feature dimension (*F* = 60 for MFCC, PLP, and FBANK), and *T* is the sequence length.

In this study, Adam Weight Decay Regularization optimizer was used in Resnet, and the learning rate was set to 0.001. The attenuation factor was 1.0, and the number of training sessions was 21.

### 4.3. Multi-task ensemble learning method

In the front-end of the multi-task ensemble learning method, nine speaker embeddings with nine task stimuli were extracted from three acoustic features. The symbolic marks of speaker embeddings are shown in Table 4. The acoustic features can be MFCC, PLP, and FBANK. The types of speaker embedding extractors in pre-training can be i-vector, TDNN x-vector, and Resnet x-vector. Speaker embeddings are extracted according to the speaker embedding extractors. In the back-end part, *Speaker Embeddings*(*j*) and ${\text{p}}_{\left(j\right)}^{i}$ represent speaker embeddings of the *jth* speech task and the SVM classification result of the *jth* speech task from the *ith* (*i* = 1, 2, …, *n*) subject, respectively. Then, all the training set results are spliced and transposed into the matrix. The same operation is performed for the testing set, and the results of this fold are obtained by using MLP.

**TABLE 4:** The denotation of speaker embeddings.

Denotation	Description
R_m	Resnet x-vectors based on MFCC
R_p	Resnet x-vectors based on PLP
R_f	Resnet x-vectors based on FBANK
T_m	TDNN x-vectors based on MFCC
T_p	TDNN x-vectors based on PLP
T_f	TDNN x-vectors based on FBANK
I_m	I-vectors based on MFCC
I_p	I-vectors based on PLP
I_f	I-vectors based on FBANK

### 4.4. Combination of different Resnet x-vectors in multi-task ensemble learning method

**FIGURE 3:**
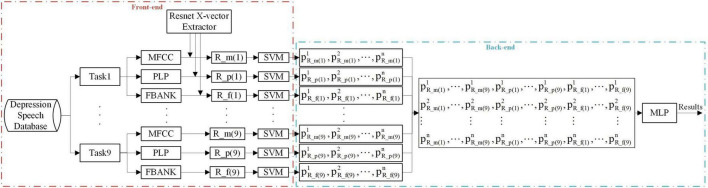
Resnet x-vector fusion of the proposed depression detection system. n denotes the number of the training subjects.

This study also combined different Resnet x-vectors in our proposed multi-task ensemble learning method. Resnet x-vectors based on different speech features contain different acoustic information, which may play a complementary role in depression recognition. Figure 3 shows that the classification results of three Resnet x-vectors (R_m, R_p, and R_f) on the training partition using SVM are fused into new features in nine tasks, and MLP is carried out to train the optimal model on the training set.*p^i^R*_*m*(*j*), *p^i^R*_*p*(*j*), and *p^i^R*_*f*(*j*) represent the SVM classification result of the *jth* speech task from the *ith* (*i* = 1, 2, …, *n*) subject based on R_m, R_p, and R_f, respectively. Although Figure 3 shows the fusion process of three Resnet x-vectors, the experiment also carries out fusion cases of two Resnet x-vectors. Additionally, the figure only shows the result of one test fold; the final result is the average of 100 repetitions of 10-fold cross-validation.

## 5. Experimental results

Our experiments have done the following work: In Section “5.1. Results of nine speaker embeddings for depression detection,” we use SVM and RF to compare the performance of nine speaker embeddings in nine tasks. We analyze the performance difference of the Resnet x-vector extractor compared with the TDNN x-vector extractor and the i-vector extractor, the impact of different acoustic features on the three speaker embedding extractors, and the impact of different speech task types and emotional valences on speaker embeddings. In Section “5.2. Results of multi-task ensemble learning methods with speaker embeddings for depression detection,” we compare the performance of our multi-task ensemble learning method and the other two literature methods in nine speaker embeddings. Moreover, the best effect is obtained by fusing Resnet x-vectors based on different features in the integrated learning method and then compared with the proposed literature studies.

In order to fully evaluate the performance of multiple speaker embeddings and ensemble learning methods in depression detection, we used accuracy, F1-D, and F1-H as performance metrics. F1-D and F1-H are F1 scores of depressed and healthy classes, respectively. For the binary problem in this paper, the four categories in the confusion matrix are True Positive (TP), False Negative (FN), False Positive (FP), and True Negative (TN). The accuracy, F1-D, and F1-H could be calculated as follows.


(2) $$a\,c\,c\,u\,r\,a\,c\,y=\frac{T\,P+T\,N}{T\,P+F\,N+F\,P+T\,N}$$

(3) $$F\,1-D=\frac{2\times T\,P}{2\times T\,P+F\,P+F\,N}$$

(4) $$F\,1-H=\frac{2\times T\,N}{2\times T\,N+F\,P+F\,N}$$

Besides, 100 repetitions of 10-fold cross-validation were employed to examine the algorithm’s performance. A total of 536 subjects (310 depressed and 226 normal) in the depression speech dataset were divided into 10 non-overlapping folds according to the proportions of the two classes. Six folds were 54 subjects (31 depressed and 23 normal), and the four folds were 53 subjects (31 depressed and 22 normal). We used ninefolds for training and the remaining fold for testing. This way, the same utterance would not appear in two different folds. The KFold function of the Scikit-learn toolbox (Pedregosa et al., 2011) (sklearn) was performed to partition the training and testing sets. The result of each repetition was an average of 10 test folds. In order to assess the generalizability of our approach, the final result was the average of 10-fold cross-validation for 100 times with different random_state (Mobram and Vali, 2022) has used this experimental scheme.

### 5.1. Results of nine speaker embeddings for depression detection

After the implementation of the front-end part of the experimental framework in Figure 1, nine speaker embeddings in nine speech tasks were obtained. Two classifiers, SVM and RF, were used to evaluate the depression recognition performance of nine speaker embeddings comprehensively. We trained SVM classifiers with a Gaussian kernel function and tuned the SVR hyper-parameters. Similarly, n_estimators, which represented the number of trees in the forest, were optimized when training RF classifiers. Concerning the experiments of speaker embeddings on each task, the training partition was used to train models, and the results were calculated on the testing partition. The experiments followed the 10-fold cross-validation rule and were repeated 100 times with different randomizations. The accuracies of nine speaker embeddings under nine tasks using SVM and RF were reported in Table 5. The detailed meanings of the nine speaker embedding nicknames in this table are shown in Table 4. We also calculated the corresponding F1-D and F1-H, but they were too long to be listed. However, they would be used in the subsequent comparison of the algorithm’s performance.

**TABLE 5:** Accuracy comparison of nine speaker embeddings under nine speech tasks using SVM or RF classifier.

SVM	I_m	I_p	I_f	T_m	T_p	T_f	R_m	R_p	R_f
Task1	58.89%	57.71%	57.31%	62.06%	61.26%	62.45%	68.58%	60.67%	67.59%
Task2	61.46%	60.47%	60.67%	62.65%	59.29%	64.62%	63.83%	60.87%	62.45%
Task3	64.62%	65.81%	63.44%	66.01%	67.39%	68.38%	67.79%	62.06%	65.02%
Task4	70.36%	72.33%	67.79%	71.74%	71.34%	74.51%	70.75%	71.74%	71.34%
Task5	57.71%	58.30%	62.25%	67.39%	62.45%	62.85%	65.81%	62.65%	62.06%
Task6	57.71%	60.08%	59.68%	62.06%	61.66%	60.47%	64.82%	60.67%	60.47%
Task7	60.67%	62.25%	60.08%	61.46%	62.06%	61.07%	63.04%	59.88%	65.42%
Task8	62.85%	58.89%	58.70%	59.29%	62.25%	59.29%	64.23%	60.47%	62.06%
Task9	64.43%	61.66%	61.86%	59.68%	59.88%	59.09%	64.23%	61.26%	64.23%
RF	I_m	I_p	I_f	T_m	T_p	T_f	R_m	R_p	R_f
Task1	61.07%	60.28%	58.50%	61.66%	63.04%	61.26%	66.80%	59.29%	65.81%
Task2	60.28%	61.26%	59.68%	60.67%	59.88%	59.68%	64.23%	60.47%	60.67%
Task3	63.64%	62.85%	62.45%	66.40%	67.59%	66.60%	64.43%	62.25%	64.23%
Task4	66.21%	67.98%	64.52%	70.95%	73.72%	72.33%	68.18%	67.79%	68.58%
Task5	58.89%	60.28%	60.28%	62.85%	61.07%	63.83%	63.04%	60.47%	61.07%
Task6	60.28%	59.09%	61.07%	63.44%	58.70%	58.89%	64.82%	59.68%	59.29%
Task7	61.86%	59.49%	60.08%	61.46%	63.64%	61.66%	66.40%	62.85%	66.01%
Task8	59.49%	57.31%	61.07%	59.09%	60.47%	59.49%	64.03%	59.49%	62.25%
Task9	60.67%	60.89%	62.25%	58.30%	60.47%	58.50%	64.43%	61.66%	63.24%

#### 5.1.1. The effects of different speaker embedding extractors on depression detection system

**FIGURE 4:**
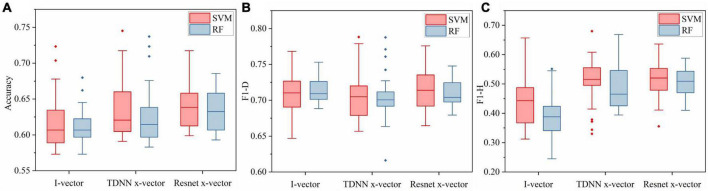
The result comparison of speaker embeddings based on three extractors in nine speech tasks between SVM and RF. **(A)** Accuracy boxplot. **(B)** F1-D boxplot. **(C)** F1-H boxplot.

Figure 4 showed classification accuracy, F1-D, and F1-H of speaker embeddings based on three extractors and the performance differences between SVM and RF. This boxplot was drawn by the results of speaker embeddings under different extractors, as described in Section “5.1. Results of nine speaker embeddings for depression detection.” For instance, the accuracy boxplot under the i-vector extractor using SVM in Figure 4A was made based on all results of I_m, I_p, and I_f under nine tasks in Table 5.

The accuracies shown in Figure 4A indicated that the Resnet x-vector extractor provided the best scores, followed by the TDNN x-vector extractor and the i-vector extractor in both SVM and RF. In detail, regardless of whether SVM or RF was used, the upper limit, median and lower limit of the Resnet x-vector extractor were highest, while those of the i-vector extractor were lowest. Although the maximum accuracy of TDNN x-vectors in SVM reached 74.51%, this number was judged as an outlier based on the overall distribution of the boxplot. Additionally, it clearly showed that the box of Resnet x-vectors was overall above the other two. Figure 4B, F1-D of Resnet x-vectors and i-vectors were close, while TDNN x-vectors were slightly inferior. Figure 4C showed that the ranking of F1-H of the three extractors was consistent with that of accuracies.

As could be seen from the results of three assessment criteria under the two classifiers, the Resnet x-vector extractor outperformed the TDNN x-vector extractor, which indicated that the ability of upstream knowledge learned by Resnet to transfer to depression screening was stronger than TDNN. Moreover, the DNN embeddings (Resnet x-vectors and TDNN x-vectors) could utilize speakers’ traits to build more effective depression models than i-vectors. The results of Egas-López et al. (2022) comparing the performance of DNN embeddings and i-vectors for depression discrimination also supported the above conclusion. It was worth noting that in the three charts of Figure 4, almost all upper limit, upper quartile, and median of the three extractors’ whole measurement indicators in SVM were higher than RF. This point was consistent with the deduction of experiments that compared classification results of SVM and RF in various i-vectors (Xing et al., 2022). Consequently, we only contrasted the results of speaker embeddings under SVM in the subsequent analysis. On the other hand, we opted for SVM to train classifiers as the back-end part of the framework displayed in Figure 1 and then integrated nine speech tasks.

#### 5.1.2. The effects of different acoustic features on depression detection system

**FIGURE 5:**
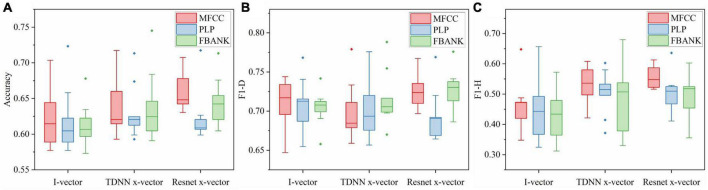
The result comparison of speaker embeddings based on different acoustic features in nine speech tasks using SVM. **(A)** Accuracy boxplot. **(B)** F1-D boxplot. **(C)** F1-H boxplot.

This part was to find out the most suitable phonetic features for each speaker embedding extractor. The accuracy, F1-D, and F1-H of three speaker embedding extractors based on MFCC, PLP, and FBANK over nine tasks using SVM were plotted in Figure 5. In terms of i-vectors, the medians of three evaluation indicators of the MFCC-based systems exceeded those of systems based on PLP or FANK, and in Figure 5A, the upper limit and upper quartile of the accuracy of MFCC i-vectors were supreme among three i-vector extractors based on different characteristics. Accordingly, MFCC was more suitable for i-vectors. In addition, (Di et al., 2021) demonstrated the effectiveness of MFCC i-vectors in the clinical diagnosis of MDD. From the comprehensive analysis of the three boxplots in Figure 5, FBANK outperformed the other feature sets in TDNN x-vectors slightly. Although the accuracies of TDNN x-vectors based on the three feature sets were similar, the median of F1-D and the upper limit of F1-H of FBANK-based systems had advantages. It could also be seen in Egas-López et al. (2022) that TDNN x-vector extractors fitted with FBANK outperformed MFCC, which our results supported. As for the Resnet x-vector extractor, it could be observed that accuracy, F1-D, and F1-H of MFCC-based systems performed better than the other two. As far as we know, there is a lack of research on the befitting phonetic features of these speaker embedding extractors. The results of our experiment can provide some reference for this problem.

**FIGURE 6:**
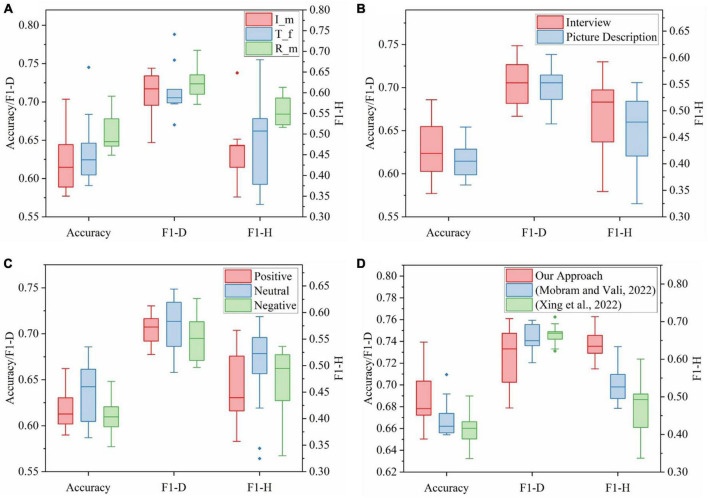
**(A)** The result comparison of each extractor based on the most matching feature set in nine speech tasks using SVM. **(B)** The result comparison of speaker embeddings in speech tasks of different genres using SVM. **(C)** The result comparison of speaker embeddings in speech tasks of different emotions using SVM. **(D)** The result comparison of three ensemble learning methods with speaker embeddings combined with nine tasks.

Since the i-vector and Resnet x-vector extractors best matched MFCC and the TDNN x-vector extractor best matched FBANK, Figure 6A showed the results of three speaker embeddings (I_m, T_f, and R_m) using SVM in nine tasks for depression classification. It was worth noting that five characteristic values of the accuracy of R_m were optimal, and its data is the most centralized. The upper limit and lower quartile of F1-D of R_m were significantly higher, and other characteristic values were not lower. The characteristic values of F1-H of R_m, except for the upper limit, were obviously better than others. As a result, R_m provided the most vital ability to recognize depression in nine tasks among nine speaker embeddings. However, the accuracy and F1-H of TDNN x-vectors were slightly better than those of i-vectors. Therefore, the performance of the three speaker embeddings was sorted from good to bad: R_m, T_f, and I_m. This conclusion could correspond to the performance ranking of three speaker embedding extractors in Section “5.1.1. The effects of different speaker embedding extractors on depression detection system.”

#### 5.1.3. The effects of different speech tasks on depression detection system

This series of analyses were conducted to investigate the influence of different genres and emotions of speech tasks on depression discrimination results of speaker embeddings. As mentioned in Table 1, there were nine tasks of the depression speech database covering two genres and three emotional valences. Table 6 integrated the accuracy of the same emotion in the same scenario in Table 5. Specifically, the accuracies of Int-Pos were the average of those of task1 and task2. The accuracies of Int-Neu were the average of those of task3 to task5. Also, the values of Int-Neg, Pic-Pos, Pic-Neu, and Pic-Neg corresponded to task6 to task9, respectively. F1-D and F1-H of six task types also performed similar operations. This operation ensured that the data volume of the six task types was the same and that the data distribution could be fairly compared through boxplots. Figure 6B and Figure 6C showed the results of nine speaker embeddings in the interview or picture description tasks and positive, neutral, or negative emotions using SVM. Moreover, the accuracy boxplots of both figures were plotted according to Table 6.

**TABLE 6:** Accuracy comparison of nine speaker embeddings under interview or picture description tasks with different emotions using SVM classifier.

Task	I_m	I_p	I_f	T_m	T_p	T_f	R_m	R_p	R_f
Int-Pos	60.18%	59.09%	58.99%	62.36%	60.28%	63.54%	66.21%	60.77%	65.02%
Int-Neu	64.23%	65.48%	64.49%	68.38%	67.06%	68.58%	68.12%	65.48%	66.14%
Int-Neg	57.71%	60.08%	59.68%	62.06%	61.66%	60.47%	64.82%	60.67%	60.47%
Pic-Pos	60.67%	62.25%	60.08%	61.46%	62.06%	61.07%	63.04%	59.88%	65.42%
Pic-Neu	62.85%	58.89%	58.70%	59.29%	62.25%	59.29%	64.23%	60.47%	62.06%
Pic-Neg	64.43%	61.66%	61.86%	59.68%	59.88%	59.09%	64.23%	61.26%	64.23%

The results of Figure 6B presented that the interview scene had more considerable fluctuations of accuracy and F1-D. However, the upper limit, median, and upper quartile of the three assessment criteria were significantly higher than the picture description scene. Even all indexes of the F1-H boxplot of the interview were superior to the picture description. Overall, interview speech performed better than picture description speech using speaker embeddings in depression detection. Although both interview speech and picture description speech were considered as spontaneous voice, we inferred from our experimental results that subjects were more likely to express their true feelings in the interview scene, and interview voice contained more information related to emotional states than picture description. This view coincides with the conclusion of Long et al. (2017).

It could be seen from Figure 6C that the accuracy, F1-D, and F1-H of neutral stimulus materials were evidently superior to positive and negative materials. Although F1-H of positive speech had no advantage over negative speech, all indexes of its accuracy were slightly higher than the negative, and five characteristic values other than the upper limit of F1-D were higher than the negative. In addition, the fluctuation of F1-D of negative speech was also the smallest. Hence, it could be concluded that neutral stimulus materials performed best, followed by positive materials and negative materials. This discovery was consistent with (Liu et al., 2017), which showed that neutral stimuli performed best among three emotional valences when using speaker embeddings for depression detection.

### 5.2. Results of multi-task ensemble learning methods with speaker embeddings for depression detection

The back-end part of Figure 1 was conducted on nine speaker embeddings, and each integrated nine speech tasks. We implemented MLP using the GridSearchCV function from sklearn, which performed grid optimization of the parameters on the training set and then applied the optimal model on the training partition to the prediction of the testing partition. Note that the result in Figure 1 was just the result of a test fold, and our method’s final result was the average of 10 test folds across 100 times.

Our approach was compared with two other ensemble methods. The first method (Mobram and Vali, 2022) was to classify speaker embeddings on nine speech tasks using cosine similarity and then a majority vote based on the results of nine tasks. The second method (Xing et al., 2022) used SVM on speaker embeddings over nine tasks and selected tasks with significant accuracy differences using paired *T*-test. Then the results of the different tasks were integrated into new features for SVM classification. The final results of these two methods were also the average of 100 repetitions of ten-fold cross-validation.

The experimental results in Table 7 indicated that three ensemble learning methods performed best on MFCC-based Resnet x-vectors, which were remarked as R_m among nine speaker embeddings, which illustrated the effectiveness of R_m in depression recognition tasks. In addition, our approach provided the best accuracy (73.94%), F1-D (76.09%), and F1-H (71.30%) on R_m with improvement by 2.99, 0.15, and 7.96% compared with (Mobram and Vali, 2022) and 4.95, 1.45, and 11.25% over (Xing et al., 2022) on three assessment criteria. Figure 6D was drawn according to the data in Table 7, reflecting the performance of three methods over 9 speaker embeddings. It could be seen that the upper limit, median, and upper quartile of the accuracy of our method were higher than those of the rest two methods. Although F1-D of our approach was slightly lower than others, all indexes of F1-H of our approach were far superior to others. On the whole, the ensemble learning method we proposed performed well.

**TABLE 7:** Performance comparison of three methods with speaker embeddings combined with nine tasks.

Speaker embeddings	Accuracy			F1-D			F1-H		
	**Our approach**	**Mobram and Vali, 2022**	**Xing et al., 2022**	**Our approach**	**Mobram and Vali, 2022**	**Xing et al., 2022**	**Our approach**	**Mobram and Vali, 2022**	**Xing et al., 2022**
I_m	65.04%	67.39%	65.22%	67.88%	75.91%	74.79%	61.61%	49.54%	43.95%
I_p	67.83%	65.42%	66.01%	71.25%	74.07%	76.24%	63.37%	48.07%	40.28%
I_f	67.23%	65.61%	63.45%	69.82%	74.56%	74.76%	64.07%	46.95%	33.69%
T_m	67.19%	67.00%	66.20%	73.31%	73.62%	74.21%	57.44%	55.94%	51.00%
T_p	70.36%	66.21%	67.39%	73.40%	73.73%	75.63%	66.52%	52.63%	50.75%
T_f	69.96%	69.17%	66.63%	76.03%	75.55%	74.89%	59.79%	58.29%	50.15%
R_m	73.94%	70.95%	68.99%	76.09%	75.94%	74.64%	71.30%	63.34%	60.05%
R_p	71.15%	65.42%	63.24%	74.74%	72.78%	73.12%	66.36%	52.57%	41.88%
R_f	67.25%	65.81%	65.05%	70.25%	72.05%	73.30%	63.44%	55.98%	49.28%

#### 5.2.1. Combining different Resnet x-vectors in multi-task ensemble learning method

Since the advantages of Resnet x-vector extractors compared to TDNN x-vector and i-vector extractors had been explained in Section “5.1.1. The effects of different speaker embedding extractors on depression detection system,” we would fuse different Resnet x-vectors (R_m, R_p, or R_f) in the multi-task integrated learning method as shown in Figure 3. The experiment was to examine the effect of this fusion on the performance of depression detection. It was not difficult to find from Table 8 that when R_m was eliminated from R_m + R_p + R_f, the accuracy, F1-D, and F1-H were reduced by 1.77, 1.19, and 3.17%, respectively. MFCC simulates the audio system of the human ear, which can suppress high-frequency signals, and reduce the interference of environmental noise. Therefore, R_m (MFCC-based Resnet x-vectors) did well in our experiment and provided a significant performance boost during the integration process. Moreover, the results in Table 8 indicated that R_m + R_p provided the highest accuracy (74.72%), F1-D (76.90%), and F1-H (72.05%), with the improvement of 0.78, 0.81, and 0.75% compared with R_m, and with the improvement of 3.57, 2.16, and 5.69% compared with R_p. PLP uses a linear prediction autoregressive model to obtain cepstrum coefficients, which is different from the compression coefficient used by MFCC. PLP also has good noise robustness. The combination of R_m and R_p should have better noise robustness than speaker embeddings before the combination. In this experiment, the speaker embeddings for depression recognition were based on the pre-trained model of out-domain data. It is very important to alleviate the interference of noise for the performance of the depression recognition model.

**TABLE 8:** Performance of ensemble fusion system of Resnet x-vectors based on different feature sets.

Ensemble fusion	Accuracy	F1-D	F1-H
R_m + R_p	74.72%	76.90%	72.05%
R_m + R_f	73.76%	75.42%	71.76%
R_p + R_f	69.60%	72.60%	65.78%
R_m + R_p + R_f	71.37%	73.79%	68.95%

#### 5.2.2. Comparison with other proposed methods on the depression speech dataset

This section compares the proposed multi-tasking integrated learning method incorporating different Resnet x-vectors with other literature studies, as shown in Table 9. Since the depression speech dataset used in this study was collected by the Gansu Provincial Key Laboratory of Wearable Computing, the results in Table 9 were obtained by implementing the methods in other papers based on this data. Note that the depression dataset was fairly divided into ten portions. Nine portions were for training, and one portion was for testing, which was unseen data. The final result of each method was the average of 100 repetitions of 10-fold cross-validation.

**TABLE 9:** Performance comparison of other literature studies on the depression speech dataset.

Method	Accuracy	F1-D	F1-H
Giannakopoulos, 2015	67.98%	74.77%	56.22%
Di et al., 2021	66.40%	72.93%	55.73%
Egas-López et al., 2022	68.18%	75.42%	54.90%
Xing et al., 2022	71.89%	77.27%	63.08%
Our proposed system	74.72%	76.90%	72.05%

Our result in Table 9 is the best one of the completed outcomes: the fusion of the MFCC-based Resnet x-vectors and the PLP-based Resnet x-vectors in the multi-task ensemble learning method, with an accuracy of 74.72%, F1-D of 76.90%, and F1-H of 72.05%. Furthermore, our system increases accuracy by 6.74%, F1-D by 2.13%, and F1-H by 15.83% compared to Giannakopoulos (2015), which classified short-term and mid-term voice features from depressed and normal subjects using the SVM classifier with RBF kernel. Also, we improved accuracy by 8.32%, F1-D by 3.97%, and F1-H by 16.32% compared to Di et al. (2021), which only used MFCC i-vectors for depression detection and improved accuracy by 6.54%, F1-D by 1.48%, and F1-H by 17.15% compared to Egas-López et al. (2022) which used pre-trained DNN embeddings based on FBANK for SVM classification. Finally, compared to Xing et al. (2022), which was the hierarchical classification method of combined i-vectors based on several speech features that we published earlier, our accuracy is improved by 2.83% and F1-H by 8.97%, while F1-D is slightly lower.

In general, compared with other literature methods, the accuracy of our method has been improved to some extent, and F1-D, which presents the classification performance of the depressed class, also maintains a reasonable level. Particularly, F1-D, which shows the classification performance of the healthy class, has been significantly improved. This impressive result shows the effectiveness of our proposed method on the gender-independent depressive speech dataset.

## 6. Conclusion and future works

In order to find the optimal speaker embeddings for depression recognition, this paper compared the performance of three speaker embedding extractors based on different acoustic feature sets for depression detection in a multi-task depression speech database. The comprehensive performance of the new state-of-art Resnet x-vector extractor applied to depression recognition for the first time is better than that of the TDNN x-vector extractor and i-vector extractor, indicating that it can extract more depression-related information than the other two. Finally, nine speaker embeddings on three extractors (Resnet x-vector extractor, TDNN x-vector extractor, and i-vector extractor) based on MFCC, PLP, and FBANK were obtained. We concluded that MFCC was suitable for the i-vector extractor, FBANK for the TDNN x-vector extractor, and MFCC for the Resnet x-vector extractor. Moreover, MFCC-based Resnet x-vectors provided the best recognition among nine speaker embeddings.

Since our depression speech dataset consisted of nine speech tasks covering two genres (interview and picture description), and three emotional valences (positive, neutral, and negative), we explored the effects of different scenes and different emotional stimuli on depression recognition. The conclusion is that the difference in speech information between the two types of subjects in the interview task is more significant than that in the picture description task. The effect of neutral stimulus materials is better than that of positive and negative materials.

To make full use of the information from different scenes and emotions, we designed a multi-task ensemble learning method using speaker embeddings on the depression speech dataset containing nine tasks. The accuracy and F1-H of our method were significantly better than that of the other two literature studies, and F1-D maintained a similar level. In addition, the MFCC-based Resnet x-vectors among nine speaker embeddings performed best in our proposed integration approach. Our multi-task ensemble learning method based on R_m + R_p achieved best results than other literature studies using the depression speech database, indicating that MFCC-based Resnet x-vectors and PLP-based Resnet x-vectors were complementary in depression recognition, and information from 9 speech tasks was also utilized in the integrated system.

In this study, we used the out-domain dataset to train the pre-trained model to alleviate the problem of insufficient data volume in deep learning. We are also constantly collecting the depression speech dataset to expand the data volume. Then we will consider using the augmented in-domain dataset for pre-training to improve the depression recognition performance further.

## Data availability statement

The data analyzed in this study is subject to the following licenses/restrictions: Data involves privacy and has not been disclosed. Requests to access these datasets should be directed to ZL, liuzhenyu@lzu.edu.cn.

## Ethics statement

The studies involving human participants were reviewed and approved by the Tianshui Third People’s Hospital. The patients/participants provided their written informed consent to participate in this study.

## Author contributions

ZL, HY, and BH were responsible for the entire study, including study concepts and study design. ZL and HY contributed to the experimental paradigm design and wrote the manuscript. GL, QC, ZD, and LF helped collect data. ZY helped perform the analysis with constructive discussions. All authors agreed to be accountable for the content of the work.
